# Osteoporosis: “A risk factor for periodontitis”

**DOI:** 10.4103/0972-124X.55841

**Published:** 2009

**Authors:** Rekha Rani Koduganti, Chandana Gorthi, P. Veerendranath Reddy, N. Sandeep

**Affiliations:** *Professor and H.O.D, Department of Periodontics, Panineeya Mahavidhyalaya Institute of Dental Sciences and Research, Kamala Nagar, Dilsukhnagar, Hyderabad - 500 060, India*; 1 *Sr. Lecturer, Department of Periodontics, Panineeya Mahavidhyalaya Institute of Dental Sciences and Research, Kamala Nagar, Dilsukhnagar, Hyderabad - 500 060, India*; 2 *Reader, Department of Periodontics, Panineeya Mahavidhyalaya Institute of Dental Sciences and Research, Kamala Nagar, Dilsukhnagar, Hyderabad - 500 060, India*

**Keywords:** Osteoporosis, osteopenia, periodontal attachment loss, prevention, risk factors

## Abstract

Aging is one of the major health challenges today. Most of the diseases related to aging, lead to significantly increased morbidity and mortality and higher public expenditure of funds. The interconnection between socio-economic conditions and social vulnerability is reflected in precarious states of health with prominence of high rates of osteoporosis and periodontal disease.Both these diseases have been highlighted in public health because of the impact caused by bone fracture and tooth loss. Thus, the elderly could help live a healthier and more meaningful life with the prevention of these diseases.

## INTRODUCTION

Osteopenia and osteoporosis are systemic skeletal diseases, characterized by low bone mass, with a consequent increase in bone fragility and susceptibility to fracture.[1]

According to World Health Organization (WHO) osteoporosis is considered to be present when the bone mineral density (BMD) is 2.5 standard deviations below the young normal. Osteopenia is defined as bone density levels 1 standard deviation and 2.5 standard deviations below normal.[2]

The prevalence of this relatively silent disease is very high.[3] Future projections indicate a three-fold increase in osteoporosis related hip fractures.[4]

Worldwide approximately one-third of women aged 60-70 years and two-thirds of women aged 80 years and above have osteoporosis.[5]

The clinical importance of systemic bone loss as a contributory factor to alveolar bone loss and subsequent loss of teeth requires to be studied extensively. Moreover the possibility osteoporosis and periodontal diseases could be related because they share common etiological agents, which could affect or modulate their natural history, should be looked into.[6,7]

## ASSESSMENT OF OSTEOPOROSIS

Conventional radiographs are not sensitive enough to diagnose osteoporosis, until the total bone density has decreased by 50%. The most widely used techniques for assessment of bone mineral density are dual-energy X-ray absorptiometry(DXA) and quantitative computed tomography.[8,9]

Dual energy X-ray absorptiometry is the most precise and the diagnostic measure of choice as quantitative computed tomography though being more sensitive, causes greater radiation exposure.[9]

## RISK FACTOR FOR OSTEOPOROSIS

The risk factors are enumerated in the [Table 1].[10]

**Table 1:** Risk factors for osteoporosis[10]

Non-modifiable risk factors	Modifiable risk factors
Age	Sex hormone insufficiency
Race	Calcium intake
Sex	Vitamin D intake
Family history of osteoporosis/fracture	Weight
Early menopause	Physical activity
	Cigarette smoking
	Chronic
	Glucocorticoid use

### Age

Bone mass changes in a person's lifetime can be categorised into the following three phases-growth, consolidation and involution. Peak bone mass is accumulated in the growth phase wherein 90% of the ultimate bone mass is deposited. This is followed by consolidation phase which lasts for 15 years. It is in this phase that the influence of exercise on building peak bone mass should be emphasized. The involution phase starts between ages 35 and 40 in both the sexes, with acceleration of bone loss within a decade after menopause in women.[11] The proportion of women with normal bone density declines sharply with increasing age.[12,13]

### Genetics

Women are more susceptible to osteoporosis than men. However, osteoporosis in men, particularly at an older age is an important health problem in the elderly.[14]

Genetics play an important role in regulating bone density, skeletal geometry, and bone turn over as well as contributing to the pathogenesis of the osteoporotic fracture as evidenced by hereditary studies.[15,16]

### Hormones

Gonadal hormones are the most important influence on bone loss in women. The onset of menopause and subsequent estrogen deficiency can affect the rate of bone loss[17–19] Rapid bone loss can be prevented by hormone replacement therapy.[18,20–22]

For regulation of bone mineral density in men, testosterone is considered to be of primary importance, though estrogen also appears to play a role at a later age in establishing peak bone mass and maintaining bone mineral density.[23–26]

### Nutrition

Calcium intake is very important for skeletal growth and peak bone mass development.[27] Increasing the intake of milk in adolescents has been shown to improve bone mineralization.[28] Calcium supplementation in post-menopausal women with low habitual dietary calcium intake may be effective in reducing bone loss.[29,30]

The body weight history of women with anorexia nervosa is the most important predictor of the presence of osteoporosis.[31] Therefore, all persons with eating disorders remain at increased risk for osteoporosis.

Vitamin D is essential for optimal absorption of calcium. Deficiency in vitamin D contributes to osteoporosis and fractures through its effects on bone fragility and impaired muscle strength.[32]

High consumption of fruits and vegetables, and the resulting high intake of dietary alkali have beneficial effects on bone mineral density.[33]

### Behavioural aspects

Cigarette smoking is associated with low bone mineral density as well as increased risk for fractures in men and women. This effect will only slowly diminish after a person stops smoking.[34] Heavy alcohol consumption has been shown to depress osteoblast function and thus decrease bone formation.

Lack of physical activity is associated with an increased risk of osteoporosis, whereas weight bearing and muscular activity stimulate bone formation and increase bone mass.[35]

Low body weight and weight loss are both established risk factors for low bone mass and an increased rate of bone loss.[36–38]

Bone loss can also be induced by medications, the most important of which are glucocorticoids.[39]

## OSTEOPOROSIS-PREVENTION STRATEGIES

It is important to identify the risk factors for individual patients and develop preventive strategies for them. There are general principles and recommendations for preventionformulated by the National Osteoporosis Foundation.

- All women should be counselled on the risk factors.
- An evaluation of bone mineral density should be performed on all post -menopausal women who present with fractures to determine the diagnosis and disease severity.
- Bone mineral density testing is recommended for all post-menopausal women younger than 65 years, who have one or more risk factors for osteoporosis in addition to menopause.
- Bone mineral density testing is recommended for all women 65 years and older regardless of additional risk factors.
- All diagnosed patients are counselled to obtain an adequate dietary intake of calcium.
- Regular weight bearing and muscle strengthening exercises to reduce the risk of falls and fractures are recommended.
- Patients should be advised against smoking and smoking cessation should be implemented. Alcohol intake should be at a moderate level (about one drink per day for women and two drinks per day for men).
- All post-menopausal women who present with hip fractures or vertebral fractures should be considered candidates for osteoporosis treatment.

Pharmacological options for osteoporosis prevention and treatment are, Hormone Replacement Therapy, Alendronate, and Raloxifene for prevention, and Calcitonin for treatment.[40]

Clinicians, including dentists, should inform and motivate the public to make and sustain life style changes relating to exercise, diet, tobacco, and alcohol use.

The National Osteoporosis Foundation as well as the National Academy of Sciences recommends a daily intake of 1200 mgms of dietary calcium and 400-800IU of vitamin D.

Tobacco use should be discouraged and current smokers should be encouraged to quit on their own or participate in smoking cessation programmes. Counselling and treatment should be offered to patients with excessive alcohol consumption as part of the life style modifications to prevent osteoporosis.[41]

The beneficial effects of physical activity and weight bearing exercises have been well documented.[42–44]

### Pharmacological intervention

Several pharmacological agents are available to increase bone mineral density and therefore treat or prevent osteoporosis. They include hormone replacement therapy, bisphosphonates, calcitonin, selective estrogen receptor modulators, parathyroid hormone or combination of these medications.

There is sufficient evidence in the literature to demonstrate that depending on the drug and the patient population, treatment reduces the risk of vertebral fractures by 30-65% and non-vertebral fractures by 46-53%.[45]

### Hormone replacement therapy

Rapid loss of bone density is observed because of estrogen deficiency in the early post-menopausal years. The rationale for HRT is to delay this bone loss. Estrogen therapy can inhibit osteoclast formation and function and can also extend the lifespan of osteoblasts and osteocytes.[46]

In a randomized clinical trial as part of the women's health initiative trial, women were randomly assigned to receive conjugated estrogens, with or without a progestin, the reduction in hip fractures was 33%.[47] HRT increased total hip bone density and reduced the risk of fractures at the hip, vertebrae and wrist.[48]

Discontinuation of estrogen results in measurable bone loss, although it is not certain whether discontinuation results in a greater fracture risk than continuation.[49]

Recently, concern has been raised about the non-skeletal risks associated with long term use of estrogen. Evidence of an increased risk of breast cancer and of cardiovascular outcomes during the course of the estrogen plus progestin trial of the women's health initiative prompted early termination of this trial in 2002.[47]

This has led to a re-evaluation of the role of HRT in the treatment and prevention of osteoporosis. HRT should not be recommended for prevention of osteoporosis in post-menopausal women, unless the woman are at a significant risk of osteoporosis, and other osteoporosis medications are unable to be considered.[50]

It is therefore important that women discontinuing HRT receive appropriate screening for their risk for complications of osteoporosis and should be counselled regarding alternative forms of therapy to prevent fracture.[51]

### Selective estrogen receptor modulators

They were developed to provide the benefits of estrogen therapy without its unwanted side effects. Their mechanism of action such as that of raloxifene is similar to that of the estrogens.[52]

Reduction in fractures was observed in the first year of treatment but no effect was found on the risk of non-vertebral fractures. Adverse effects include hot flashes and cramps. Similar to estrogen therapy, an increase in the incidence of deep vein thrombosis was observed.[53]

New selective estrogen- receptor modulators are being researched and may be available in the near future.

### Bisphosphonates

They are analogues of pyrophosphonate and bind selectively to bone mineral. During bone resorption they are taken up by the osteoclast, resulting in osteoclast de-activation and apoptosis'. Bone resorption is suppressed followed by a secondary mineralization resulting in increased bone mass, improving bone strength and a reduction in fractures.[54]

Bisphosphonates are often considered the first-line therapy for the treatment of post-menopausal osteoporosis. They are the most widely prescribed anti-resorptive agents. Randomized trials of alendronate and risedronate, two second generation bisphosphonates, demonstrated increased bone mineral density in post-menopausal women with osteopenia or osteoporosis. In women with osteoporosis a reduction in the incidence of hip, vertebral and non-vertebral fractures of nearly 50% was found. This effect was noted early in therapy.[55–58]

The occurrence of osteonecrosis of the jaws with the use of intravenous bisphosphonates is of concern to the dental community. After initial observations by Wang at the University of California, San Francisco, Marx and Migliorati reported similar findings in a new set of patients.[59–62]

Osteonecrosis of the jaws occurs more commonly in the mandible but has also been reported in the maxilla, and appears to be highly associated with periodontitis, other oral infections, and extraction of the affected teeth in majority of the reported cases. In addition the signs and symptoms that may occur before the appearance of clinically evident osteonecrosis include changes in the health of the periodontal tissues, non-healing mucosal ulcers, loose teeth and unexplained soft tissue infection. The role of oral bisphosphonates in osteonecrosis of the jaw needs to be further evaluated.

### Calcitonin

Calcitonin is an inhibitor of osteoclast activity. Both Nasal and subcutaneous calcitonin are available for treatment of post-menopausal osteoporosis. Treatment of women with osteoporosis with nasal calcitonin has been shown to reduce the incidence of vertebral fractures in a single randomized study, by 33% when compared to placebo.[63]

### The systemic oral connection

Periodontitis is an inflammatory disease characterized by loss of connective tissue and alveolar bone. Like osteoporosis, it is a silent disease causing symptoms until late in the disease process when mobile teeth, abscesses and tooth loss may occur.

Both osteoporosis and periodontal disease share many risk factors and since both are bone resorptive diseases it has been hypothesized that osteoporosis could be a risk factor for progression of periodontal disease.[1]

## RELATION BETWEEN SYSTEMIC BONE MINERAL DENSITY AND ORAL BONE MINERAL DENSITY

Most studies reported to date concerning this relationship are cross-sectional studies using different populations and different methods to assess BMD.

Kribbs *et al.*,[64] was the first to address the relationship in osteoporotic women in a study assessing total body calcium by neutron activation analysis. An association was found with mandibular density when measured by quantitative analysis on intraoral radiographs.

In another study a comparison of 85 osteoporotic women and 27 normal women was made. The osteoporotic group had less mandibular bone mass and density and a thinner cortex at the gonion than the normal group.[65]

In another study done by the same author on 85 osteoporotic post-menopausal women the total body calcium, bone mass at radius and bone density at the spine correlated with mandibular mass.[66]

A cross-sectional study in a group of 50 normal women aged 20-90 years was done and it was inferred that the mandibular bone mass correlated with the bone mass at spine and wrist.[67]

Von Wowern *et al.,*[68] in a study of 12 osteoporotic subjects with the history of fractures found less mandibular bone mineral content as measured by dual photon absorptiometry than in 14 normal women. In a longitudinal study of 69 women receiving hormone replacement therapy, lumbar spine BMD was assessed by dual photon absorptiometry. During the observation period of an average of five years a positive effect of estrogen replacement therapy on the bone mass of the mandible and the lumbar spine was observed. Different estrogen regimens resulted in different increases in bone mass.[69]

Streckfus *et al.*,[70] used quantitative measurements of vertical bitewing and hand radiographs in patients with active periodontitis. The results of this study showed that post-menopausal women on estrogen therapy had more alveolar bone loss, more missing teeth and reduced alveolar and second metacarpal bone density than premenopausal women. Alveolar bone densities were also strongly correlated to second metacarpal densities.

In a study of both maxilla and mandible 41 dentate Caucasian women aged 20-78 years were evaluated using quantitative intraoral radiography and systemic bone densities determined by dual energy x ray absorptiometry. The density of maxillary alveolar process bone was significantly related to the density of the mandibular alveolar process, lumbar spine, hip and radius in healthy women and the maxillary alveolar process bone density declined with age.[71]

Shrout *et al.,*[72] performed a study in 45 postmenopausal women who had no or only mild periodontal disease (no probing depth greater than > 5 mm) using morphologic measurements from digitized images of bitewing radiographs to correlate with lumbar and femoral BMD. The complexity of trabecular pattern weakly correlated with lumbar spine and femoral BMD.

The data gathered on most of the cross-sectional studies appears to indicate a relationship between systemic BMD and oral BMD.

## PERIODONTAL DISEASE AND OSTEOPOROSIS

The relationship of tooth loss and BMD has been studied. Several reports find a correlation while others do not. The use of tooth loss as a surrogate for periodontal disease extent, has several limitations because of other factors which could contribute to it. Therefore several cross-sectional reports have used a variety of parameters to evaluate the periodontal disease severity in subjects with decreased BMD.

In a report by Elders *et al.*,[73] lumbar BMD and metacarpal cortical thickness were compared to alveolar bone height on bitewing radiographs and clinical parameters of periodontitis. No significant relation was observed between the bone mass measurements and alveolar bone height and periodontal parameters. The mean age in this group was relatively young between 46-55 years of age which could have contributed to the lack of correlation.

In another study of 70 year old women 15 subjects with osteoporosis were compared to 21 subjects with normal BMD. No statistically significant differences were found in gingival bleeding, probing pocket depths, gingival recession or marginal bone level between the women with osteoporosis and the women with normal BMD.[74]

Von Wowern *et al.*,[68] found greater amounts of loss of attachment in osteoporotic women with a mean age of 68. Osteoporosis was assessed using bone mineral content of the mandible and forearm determined by dual photon scanning.

In a study population of 70 post-menopausal Caucasian women aged 51-78, skeletal systemic BMD was assessed by DXA. Clinical attachment loss and interproximal alveolar bone loss represented periodontal disease severity. Mean alveolar bone loss significantly correlated with systemic BMD. A trend for a correlation between clinical attachment levels and BMD was found.[75]

The cross sectional studies have limitations. No information about the diseases studied prior to the exam is available. Although both osteoporosis and periodontiits are chronic diseases it is incorrect to presume that these diseases would have been present prior to the examination, therefore to better evaluate this relationship, prospective longitudinal studies have been performed.

In a two-year longitudinal study, the alveolar bone height and density changes in 21 osteoporotic/osteopenic women, compared with 17 women with normal lumbar spine BMD were studied. The osteoporotic/osteopenic women exhibited a higher frequency of alveolar bone height loss and crestal and subcrestal density loss relative to women with normal BMD. Estrogen deficiency was associated with increased frequency of alveolar bone crestal density loss in the osteoporotic/osteopenic women. The authors concluded that osteoporosis/osteopenia and estrogen deficiency are risk factors for alveolar bone density loss in post-menopausal women with a history of periodontitis.[76]

In another study 59 moderate/advanced adult periodontitis patients and 16 non-periodontitis subjects all within five years after menopause at baseline were stratified based on serum estradiol levels. Attachment loss was assessed over a two year period and correlated to BMD and serum estradiol levels. Serum estradiol levels did not influence the percentage of sites losing attachment for either periodontitis or non periodontitis groups. The estradiol deficient group had a trend towards a higher frequency of sites with attachment loss Greater than or equal to 2 mm.[77]

The oral ancillary study of the Women's Health Initiative at the University of Alabama at Birmingham was designed to determine if there is an association between systemic osteoporosis and oral bone loss. All subjects enrolled in the study were post-menopausal females. Hipbone mineral density was confirmed with DXA. Comprehensive medical histories and examinations were linked with the results of oral examinations and quantitative digital intraoral radiography.

The intraoral techniques used in this study have been validated and are over 90% sensitive and specific in detecting small changes in bone mass and density. Standardized vertical bitewing radiographs were taken at baseline and the three-year follow-up visit. Subtraction radiography was used for the enhancement of the standardized radiographs.

Alveolar bone height was measured using Periovision software. Measurements were made on the mesial and distal aspects of posterior teeth. The patients were recalled and a similar examination including the radiographic surveys was performed every three years.

The amount of Alveolar bone loss (ABL) along the root surface over the three-year period was calculated for 58 subjects using digital subtraction radiography.

The subjects were divided into two groups, based on BMD at the hip measured at baseline. The osteoporosis group was defined as hipbone mineral density 2.5 SD below the normal as confirmed by DXA. Subjects with BMD above this level were considered the non-osteoporosis group. The subjects were also stratified based on ABL as a measure of the periodontal disease status at baseline. A subject was considered to have periodontitis when 3 mm or greater of alveolar bone height was measured at baseline. Subjects with osteoporosis presented with greater progression of ABL than subjects with no osteoporosis over the 3-year period.[1]

The subjects with periodontal disease at baseline exhibited greater amounts of ABL than subjects without periodontal disease. The greater amount of ABL was found in the group of subjects with periodontal disease and osteoporosis. When, periodontitis was present at baseline the mean ABL for patients with periodontitis and osteoporosis was 1.08 plus/minus 0.46 mm compared with 0.31 plus/minus 0.20 mm in the non-osteoporosis group. This would indicate that osteoporosis or low systemic BMD should be considered a risk factor for periodontal disease progression.

## CONCLUSION

The effects of osteoporosis on both systemic health and oral health need to be well understood. As a health care provider the dentist could serve as a pre-screener of patients with the potential for osteopenia or osteoporosis. Familiarity with the risk factors could help identify these individuals and aid in earlier diagnosis.

Although a positive association between osteoporosis and periodontal disease was found, and despite the incipient evidence linking osteoporosis and periodontitis, additional studies are needed to elucidate this topic. These might include other types of study design, possibly with intervention before menopause, with long term follow up, and investigation of oral conditions during the postmenopausal phase.

## References

[1] Geurs NC, Lewis CE, Jeffcoat MK (2003). Osteoporosis and periodontal disease progression. Periodontol 2000.

[2] WHO (1994). Assessment of fracture risks and its applications to screening for postmenopausal osteoporosis. WHO Technical Report Series.

[3] Melton LJ, Lane AW, Cooper C, Eastell R, O'Fallon WN, Riggs BL (1993). Prevalence and incidence of vertebral deformities. Osteoporos Int.

[4] Kannus P, Niemi S, Parkkari J, Palvanen M, Vuori I, Jarvinen M (1999). Hip fractures in Finland between 1970 and 1997 and predictions for the future. Lancet.

[5] International Osteoporosis Foundation (2004). Foundation IO the facts about osteoporosis and its impact.

[6] Garcia RI, Henshal MM, Krall EA (2001). Relationship between periodontal disease and systemic health. Periodontol 2000.

[7] Yoshihara A, Seida Y, Hanada N, Miyazaki H (2004). A longitudinal study of the relationship between periodontal disease and bone mineral density in community-dwelling older adults. J Clin Periodontol.

[8] Raisz LG (2005). Clinical practice: Screening for osteoporosis. N Engl J Med.

[9] Watts NB (2004). Fundamentals and pitfalls of bone densitometry using dual-energy x-ray absorptiometry (DXA). Osteoporos Int.

[10] Wehren LE (2003). The epidemiology of osteoporosis and fractures in geriatric medicine. Clin Geriatr Med.

[11] Riggs BL, Melton LJ (1986). Involutional osteoporosis. N Engl J Med.

[12] Looker AC, Johnston CC, Wahner HW, Dunn WL, Calvo MS, Harris TB (1995). Prevalence of low femoral bone density in older U.S. women from NHANES III. J Bone Miner Res.

[13] Looker AC, Orwoll ES, Johnston CC, Lindsay RL, Wahner HW, Dunn WL (1997). Prevalence of low femoral bone density in older U.S. adults from NHANES III. J Bone Miner Res.

[14] Orwoll ES (1998). Osteoporosis in men. Endocrinol Metab Clin North Am.

[15] Jin H, Ralston SH (2005). Genetics of osteoporosis. Curr Rheumatol Rep.

[16] Levi G, Geoffroy V, Palmisano G, de Vernejoul MC (2002). Bones, genes and fractures: Workshop on the genetics of osteoporosis: From basic to clinical research. EMBO Rep.

[17] Christiansen C, Christensen MS, Transbol I (1981). Bone mass in postmenopausal women after withdrawal of estrogen/ gestagen replacement therapy. Lancet.

[18] Lindsay R, Hart DM, Forrest C, Baird C (1980). Prevention of spinal osteoporosis in oophorectomised women. Lancet.

[19] Rae MH, Mole TA, Paterson CR (1991). Endogenous factors affecting bone mineral content in post-menopausal women Maturitas.

[20] Christiansen C, Christensen MS, McNair P, Hagen C, Stocklund KE, Transbol I (1980). Prevention of early postmenopausal bone loss: Controlled 2-year study in 315 normal females. Eur J Clin Invest.

[21] Lindsay R, Hart DM, Aitken JM, MacDonald EB, Anderson JB, Clarke AC (1976). Long-term prevention of postmenopausal; osteoporosis by oestrogen, Evidence for an increased bone mass after delayed onset of oestrogen treatment. Lancet.

[22] Riggs BL, Melton LJ (1992). The prevention and treatment of osteoporosis. N Engl J Med.

[23] Carani C, Qin K, Simoni M, Faustini-Fustini M, Serpente S, Boyd J (1997). Effect of testosterone and estradiol in a man with aroma taste deficiency. N Engl J Med.

[24] Falahati-Nini A, Riggs BL, Atkinson EJ, O'Fallon WM, Eastell R, Khosla S (2000). Relative contributions of testosterone and estrogen in regulating bone resorption and formation in normal elderly men. J Clin Invest.

[25] Khosla S, Melton LJ, Atkinson EJ, O'Fallon WM (2001). Relationship of serum sex steroid levels to longitudinal changes in bone density in young versus elderly men. J Clin Endocrinol Metab.

[26] Slemenda CW, Longcope C, Zhou L, Hui SL, Peacock M, Johnston CC (1997). Sex steroids and bone mass in older men: Positive associations with serum estrogens and negative associations with androgens. J Clin Invest.

[27] Heaney RP, Abrams S, Dawson-Hughes B, Looker A, Marcus R, Matkovic V (2000). Peak bone mass. Osteoporos Int.

[28] Cadoge J, Eastell R, Jones N, Barker ME (1997). Milk intake and bone mineral acquisition in adolescent girls: Randomised, controlled intervention trial. BMJ.

[29] Davies KM, Heaney RP, Recker RR, Lappe JM, Barger-Lux MJ, Rafferty K (2000). Calcium intake and body weight. J Clin Endocrinol Metab.

[30] Heaney RP, Berner B, Louie-Helm J (2000). Dosing regimen for calcium supplementation (See comment). J Bone Miner Res.

[31] Hotta N, Shibasaki T, Sato K, Demura H (1998). The importance of body weight history in the occurrence and recovery of osteoporosis in patients with anorexia nervosa: Evaluation by dual x-ray absorptiometry and bone metabolic markers. Eur J Endocrinol.

[32] Bischoff-Ferrari HA, Willett WC, Wong JB, Giovannucci E, Dietrich T, Dawson-Hughes B (2005). Fracture prevention with vitamin D supplementation: A meta-analysis of randomized controlled trials. J Am Med Assoc.

[33] Barzel US (1995). The skeleton as an ion exchange system: Implications for the role of acid-base imbalance in the genesis of osteoporosis. J Bone Miner Res.

[34] Kanis JA, Johnell O, Oden A, Johansson H, De Laet C, Eisman JA (2005). Smoking and fracture risk: A meta-analysis. Osteoporos Int.

[35] Gross GJ, Ott CD, Lindsey AM, Twiss JJ, Waltman N (2002). Postmenopausal breast cancer survivors at risk for osteoporosis: Physical activity, vigour, and vitality. Oncol Nurs Forum Online.

[36] Guthrie JR, Ebeling PR, Dennerstein L, Wark JD (2000). Risk factors for osteoporosis: Prevalence, change, and association with bone density. Medscape Womens Health.

[37] Hannan MT, Felson DT, Dawson-Hughes B, Tucker KL, Cupples LA, Wilson PW (2000). Risk factors for longitudinal bone loss in elderly men and women: The Framingham Osteoporosis Study. J Bone Miner Res.

[38] Kroger H, Tuppurainen M, Honkanen R, Alhava E, Saarikoski S (1994). Bone mineral density and risk factors for osteoporosis: A population based study of 1600 premenopausal women. Calcif Tissue Int.

[39] Cannalis E (1996). Clinical review 83: Mechanisms of glucocorticoid action in bone: Implications to glucocorticoid-induced osteoporosis. J Clin Endocrinol Metab.

[40] National Osteoporosis Foundation (1998). Physicians guide to prevention and treatment of osteoporosis.

[41] North American Menopause S (2002). Management of postmenopausal osteoporosis: Position statement of the North American Menopause Society. Menopause.

[42] Ernst E (1998). Exercise for female osteoporosis: A systematic review of randomised clinical trials. Sports Med.

[43] Feskanich D, Willett W, Colditz G (2002). Walking and leisure time activity and risk of hip fracture in postmenopausal women (see comment). J Am Med Assoc.

[44] Kemmler W, Engelke K, Lauber D, Weineck J, Hensen J, Kalender WA (2002). Exercise effects on fitness and bone mineral density in early postmenopausal women: 1-year EFOPS results. Med Sci Sports Exerc.

[45] Delmas PD, Rizzoli R, Cooper C, Reginster JY (2005). Treatment of patients with postmenopausal osteoporosis is worthwhile: The position of the International Osteoporosis Foundation. Osteoporos Int.

[46] Manoglas SC (2000). Birth and death of bone cells: Basic regulatory mechanisms and implications for the pathogenesis and treatment of osteoporosis. Endocr Rev.

[47] Rossouw JE, Anderson GL, Prentice RL, LaCroix AZ, Kooperberg C, Stefanick ML (2002). Writing Group for the Women's Health Initiative I: Risks and benefits of estrogen plus progestin in healthy postmenopausal women: Principal results from the Women's Health Initiative randomized controlled trial. J Am Med Assoc.

[48] Cauley JA, Robbins J, Chen Z, Cummings SR, Jackson RD, LaCroix AZ (2003). Women's Health Initiative I: Effects of estrogen plus progestin on risk of fracture and bone mineral density: The Women's Health Initiative randomized trial. J Am Med Assoc.

[49] Greenspan SL, Resnick NM, Parker RA (2003). Combination therapy with hormone replacement and alendronate for prevention of bone loss in elderly women: A randomised controlled trial. J Am Med Assoc.

[50] Ettinger B, Grady D, Tosteson AN, Pressman A, Macer JL (2003). Effect of the Women's Health Initiative on women's decisions to discontinue postmenopausal hormone therapy. Obstet Gynecol.

[51] Barrett-Connor E, Wehren LE, Siris ES, Miller P, Chen YT, Abbott TA (2003). Recency and duration of postmenopausal hormone therapy: Effects on bone mineral density and fracture risk in the National Osteoporosis Risk Assessment (NORA) study. Menopause.

[52] Riggs BL, Hartmann LC (2003). Selective estrogen-receptor modulators: Mechanisms of action and application to clinical practice. N Engl J Med.

[53] Delmas PD, Ensrud KE, Adachi JD, Harper KD, Sarkar S, Gennari C (2002). Multiple outcomes of Raloxifene Evaluation I: Efficacy of Raloxifene on vertebral fracture risk reduction in postmenopausal women with osteoporosis: Four-year results from a randomized clinical trial. J Clin Endocrinol Metab.

[54] Reszka AA, Rodan GA (2003). Bisphosphonate mechanism of action. Curr Rheumatol Rep.

[55] Black DM, Cummings SR, Karpof DB, Cauley JA, Thompson DE, Baner DC (1996). R randomized trial of effect of alendronate on risk of fracture in women with existing vertebral fractures. Lancet.

[56] Ensrud KE, Barrett-Connor EL, Schwartz A, Santora AC, Bauer DC, Suryawanshi S (2004). Long term extension research G. Randomized trial of effect of alendronate continuation versus discontinuation in women with low BMD: Results from the Fracture Intervention Trial long term extension (see comment). J Bone Miner Res.

[57] Harris ST, Watts NB, Genant HK, McKeever CD, Hangartner T, Keller M (1999). Effects of risedronate treatment on vertebral and non-vertebral fractures in women with postmenopausal osteoporosis: A randomized controlled trial. J Am Med Assoc.

[58] McClung MR, Geusens P, Miller PD, Zippel H, Bensen WG, Roux C (2001). Hip Intervention Program Study G. Effect of risedronate on the risk of hip fracture in elderly women. Engl J Med.

[59] Marx RE (2005). Pamidronate (Aredia) and zoledronate (zometa) induced avascular necrosis of the jaws: A growing epidemic. J Oral Maxillofac Surg.

[60] Migliorati CA (2003). Bisphosphonates and oral cavity avascular bone necrosis. J Clin Oncol.

[61] Ruggiero SL, Mehrotra B, Rosenberg TJ, Engroff SL (2004). Osteonecrosis of the jaws associated with the use of bisphosphonates: A review of 63 cases. J Oral Maxillofac Surg.

[62] Wang J, Goodger NM, Pogrel MA (2003). Osteonecrosis of the jaws associated with cancer chemotherapy (see comment). J Oral Maxillofac Surg.

[63] Chestnut CH, Silverman S, Andriano K, Genant H, Gimona A, Harris S (2000). A randomized trial of nasal spray salmon calcitonin in postmenopausal women with established osteoporosis: The prevent recurrence of osteoporotic fractures study. Am J Med.

[64] Kribbs PJ, Smith DE, Chestnut CH (1983). Oral findings in osteoporosis, Part II: Relationship between residual ridge and alveolar bone resorption and generalized skeletal osteopenia. J Prosthet Dent.

[65] Kribbs PJ (1990). Comparison of mandibular bone in normal and osteoporotic women. J Prosthet Dent.

[66] Kribbs PJ, Chestnut CH, Ott SM, Kilcoyne RF (1989). Relationship between mandibular and skeletal bone in an osteoporotic population. J Prosthet Dent.

[67] Kribbs PJ, Cestnut CH, Ott SM, Kilcoyne RF (1990). Relationship between mandibular and skeletal bone in a population of normal women. J Prosthet Dent.

[68] Von Wowern N, Klausen B, Kollerup G (1994). Osteoporosis; a risk factor in periodontal disease. J Periodontol.

[69] Jacobs R, Ghyselen J, KonincKx P, van Steenberghe D (1996). Long-term bone mass evaluation of mandible and lumbar spine in a group of women receiving hormone replacement therapy. Eur J Oral Sci.

[70] Streckfus CF, Johnson RB, Nick T, Tsao A, Tucci M (1997). Comparison of alveolar bone loss, alveolar bone density and metacarpal density, salivary and gingival crevicular fluid interleukin-6 concentrations in healthy premenopausal and postmenopausal women on estrogen therapy. J Gerontol A Biol Sci Med Sci.

[71] Southard KA, Southard TE, Schlechte JA, Meis PA (2000). The relationship between the density of the alveolar process and that of the post-cranial bone. J Dent Res.

[72] Shrout MK, Hildebolt CF, Potter BJ, Brundsen TK, Pilgram TK, Dotson M (2000). Comparison of morphological measurements extracted from digitized dental radiographs with lumbar and femoral bone mineral density measurements in postmenopausal women. J Periodontol.

[73] Elders PJ, Habets LL, Netelenbos JC, van der Linden LW, van der Stelt PF (1992). The relation between periodontitis and systemic bone mass in women between 46 and 55 years of age. J Clin Periodontol.

[74] Lundstrom A, Jendle J, Stenstrom B, Toss G, Ravald N (2001). Periodontal conditions in 70 year-old women with osteoporosis. Swed Dent J.

[75] Tezal M, Wactawski-Wende J, Grossi SG, Ho AW, Dunford R, Genco RJ (2000). The relationship between bone mineral density and periodontitis in postmenopausal women. J Periodontol.

[76] Payne JB, Reinhardt RA, Nummikoski PV, Patil KD (1999). Longitudinal alveolar bone loss in postmenopausal osteoporotic/osteopenic women. Osteoporos Int.

[77] Reinhardt RA, Payne JB, Maze CA, Patil KD, Gallagher SJ, Mattson JS (1999). Influence of estrogen and osteopenia/osteoporosis on clinical periodontitis in postmenopausal women. J Periodontol.

